# Clinical experience with the treatment of retroperitoneal vascular leiomyosarcoma originating from large veins

**DOI:** 10.1186/s12893-021-01322-z

**Published:** 2021-08-15

**Authors:** Hong-xian Zhang, Kai Wang, Peng Hong, Min Lu, Zhuo Liu, Lei Liu, Guo-liang Wang, Lu-lin Ma

**Affiliations:** 1 Department of Urology, Peking University Third Hospital, 100191 Beijing, People’s Republic of China; 2 Department of Pathology, Peking University Third Hospital, 100191 Beijing, People’s Republic of China

**Keywords:** Retroperitoneal tumor, Leiomyosarcoma, Diagnosis, Treatment

## Abstract

**Background:**

Retroperitoneal vascular leiomyosarcoma (RVLMS) is an extremely rare disease in clinical practice, and it has poor prognosis. This article is to explore the diagnosis and treatment of RVLMS and present our experience.

**Methods:**

Data of RVLMS patients were continuously collected in our hospital from August 2018 to February 2020: two males and two females with a median age of 56 (min–max = 33–61) years were included. Patients in whom paraganglioma could not be excluded were asked to take phenoxybenzamine before surgery. A multi-disciplinary team (MDT) meeting had been held and surgery was recommended. The operation procedures varied based on the tumor location, shape, and stage, and the core steps were "exposure of the retroperitoneum and tumor, identification of vital blood vessels, blocking the bloodstream, complete removal of the tumor and tumor thrombus, and release of blood flow". A Satinsky clamp was used to partially block the blood vessels. Follow-up was conveyed by revisits and phone calls.

**Results:**

One patient underwent open surgery, and three patients underwent laparoscopic surgery, one of whom underwent conversion to open surgery. The procedures were finished successfully, with a median operative time of 314.5 (min–max = 224–467) mins. The median amount of intraoperative bleeding was 550 (min–max = 200–1500) ml, and three patients had transfusion during the operation. The mass was irregular in shape, with a median maximum size of 7.45 (min–max = 4.2–10.7) cm, and the pathological examination confirmed RVLMS, which has spindle-shape, high mitotic activity and atypia. One week after the operation, the median serum creatinine level was 85 (min–max = 70–99) µmol/L. The median follow-up time was 16 (min–max = 13–21) months, and 1 case reported asymptomatic recurrence.

**Conclusion:**

Uncharacteristic manifestations and imaging features contribute to the problematic diagnosis of RVLMS. Comprehensive preoperative evaluation and careful surgical planning are essential. Multicenter research is needed in the future to reach a dominant consensus.

## Background

Vascular leiomyosarcoma (LMS) is a kind of malignant mesenchymal tumor that originates from the vascular wall's smooth muscle layer. Sixty percent of cases occur in the great retroperitoneal veins, including the inferior vena cava (IVC), renal vein (RV), and adrenal vein (AV); these cases are called retroperitoneal vascular leiomyosarcoma (RVLMS). RVLMS is characterized by smooth muscle cells extending beyond the blood vessel and spreading along the wall. The tumor progresses slowly and tends to invade surrounding tissues and metastasize within the blood circulation, leading to a worse prognosis [1]. Women are more susceptible to these tumors (male: female = 1: 4), especially those aged 40–70 [2, 3]. Because of unnoticeable symptoms in the early stage, patients usually do not visit the hospital until the presence of upper abdominal or lower back pain due to the compression of other organs by the enlarged tumor. Symptoms of venous obstruction, such as lower limb edema and sensory abnormalities, may also occur. Dyspnoea or Budd-Chiari syndrome can also occur in some severe cases due to occlusion of the trachea or hepatic vessels [4]. Moreover, confusing image presentations and complicated retroperitoneal structures contribute to the low accuracy of preoperative diagnosis. Treatment decisions in clinical practice are challenging.

RVLMS is a rare disease, accounting for less than 1/100000 malignant tumors, and no more than 500 cases have been reported in previous literature. Although there are few guidelines, surgical removal to obtain a negative margin has been indicated significantly improve tumor-specific survival [5]. Artificial blood vessel replacement or autologous liver transplantation may be considered during surgery, depending on the degree of tumor invasion. Since 2018, there have been four cases of RVLMS from different blood vessels treated in our hospital. In this article, we will review these cases and related publications to develop a better understanding of RVLMS, try to establish a clinical decision map, and provide suggestions for diagnosis and treatment in future work.

## Methods

### Patient information

**Fig. 1:**
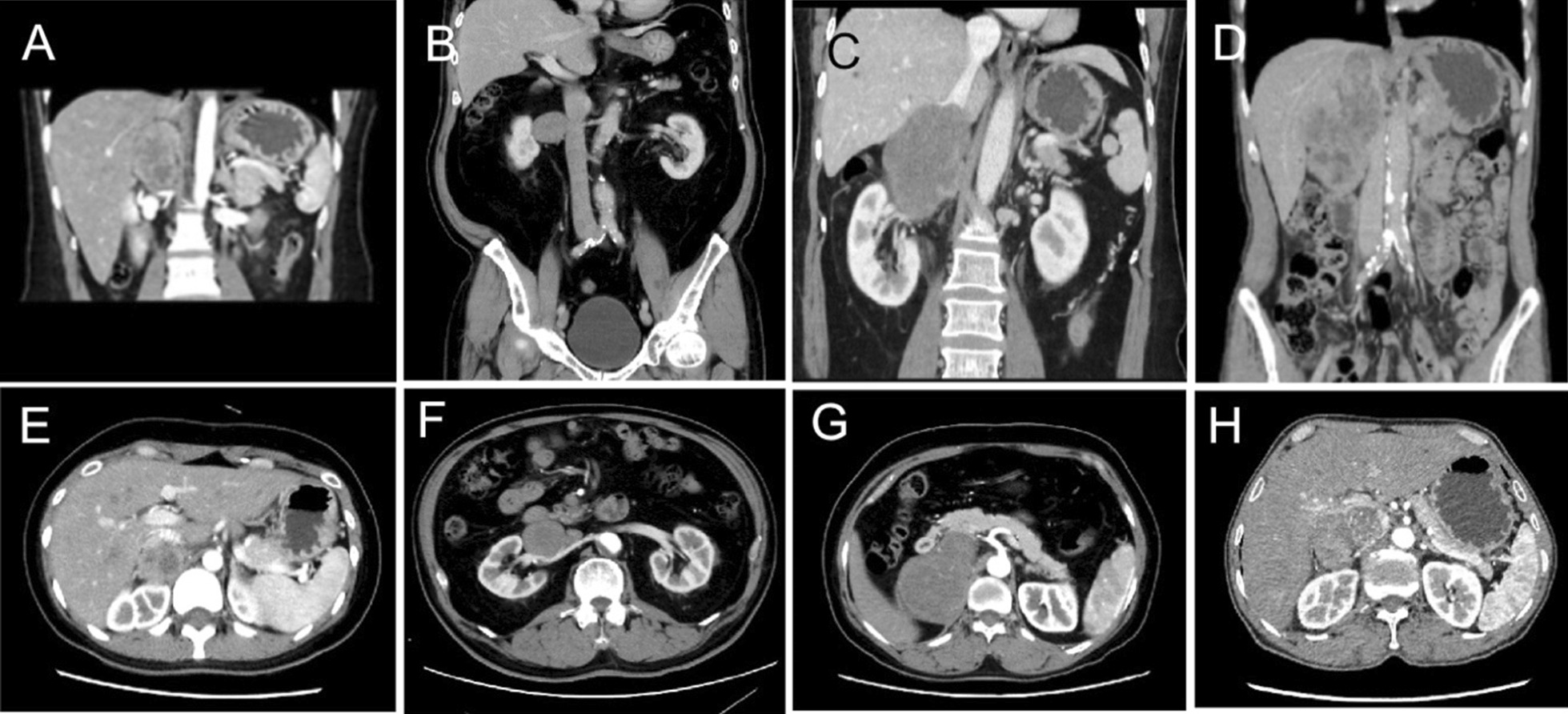
Preoperative CT images of the patients. Two patients had a mass in the right adrenal area, one patient in the right renal hilum area, and one patient in the area of the right adrenal gland anterior to the right kidney. **A/E** case 1, **B/F** case 2, **C/G** case 3; **D/H** case 4

We continuously collected all patients in our hospital who underwent surgical treatment with “retroperitoneal mass” and were confirmed to be RVLMS by pathological examination from August 2018 to February 2020.We retrospectively collected all RVLMS cases in our hospital from August 2018 to February 2020. Four patients were included: two males and two females, with a median age of 56 (min–max = 33–61) years. The main symptoms, past history, preoperative American Society of Anesthesiologists (ASA) grade and tumor size and side are presented in Table 1. Preoperative computed tomography urography (CTU) and renal and adrenal hormone determination, including blood catecholamines, 24 h 3-methyl-4-hydroxymandelic acid (VMA), adrenocorticotropic hormone (ACTH), plasma cortisol rhythm and renin-angiotensin II-aldosterone system (RAAS) function in standing and recumbent positions, were conducted. The preoperative contrast-enhanced computed tomography (CECT) images are shown in Fig. 1. A single round low-density mass was found in three patients: two were located in the right adrenal gland area (cases 1 and 3), and the other was located in the right renal hilum area (case 2). In case 4, the patient was found to have a sizeable lobular mass in front of both the right kidney and adrenal gland. In two cases, the tumor expanded into the IVC and formed a tumor thrombus; in case 4, the tumor extended to the lower edge of the second hilar region. All patients whom paragangliomas cannot be excluded were given phenoxybenzamine two weeks before the operation to prevent possible blood pressure fluctuations Three patients underwent retroperitoneal laparoscopic tumor resection; in case 2, the patient was converted to open surgery. One patient underwent open surgery. Postoperative follow-up was conducted by outpatient revisits and phone calls.

**Table 1:** Baseline information and surgical data

Item	Case 1	Case 2	Case 3	Case 4
Age	33	61	57	55
Sex	Female	Male	Female	Male
Main symptom	Abdominal pain, palpitation, headache	None	None	None
Past history	None	HTN, Hyperlipidemia	HTN	HTN
Renal and adrenal hormone tests	Normal	Normal	Normal	Normal
Size (cm)	7.2	4.2	7.7	10.7
Tumor thrombus	No	No	3 cm in IVC	7 cm in IVC
ASA	2	2	2	2
Approach	Retroperitoneal laparoscopic	Retroperitoneal laparoscopic (Conversion to open)	Retroperitoneal laparoscopic	Open
Operation time (min)	467	224	373	256
Blood loss (ml)	300	200	1500	800
Blood transfusion	800 ml RBC	0	2000 ml RBC 1400 ml Plasma	800 ml RBC
Postoperative diagnosis	IVC LMS (Middle)	RV LMS (Right)	IVC LMS (Middle)	IVC (Middle) + RV (Right) LMS
Resection margin	Negative	Negative	Negative	Negative
Tumor grading (FNCLCC)	G1	G2	G1	G2
Post-operative complication	Bleeding	None	None	None
Clavein-Dindo grading	2	–	–	–
Follow-up	16 M, AWR	16 M, AOD	13 M, AOD	21 M, AOD

*HTN* Hypertension, *IVC* Inferior vena cava, *RV* Renal vein, *LMS* Leiomyosarcoma, *AWR* Alive with recurrence, *AOD* Alive without disease

### Multidisciplinary case discussion

The diagnosis and treatment plan of soft tissue mass needs to be decided by a multidisciplinary team (MDT) meeting, which includes doctors of General Surgery, Urology, Radiology, Oncology, and Pathology. In this study, the tumor has a clear boundary, seems to have a capsule, and no distant metastasis was detected. MDT discussed and decided to choose surgery as the first choice.

### Operation procedure

The intestinal preparation was performed one day before the operation. A total of 800–1200 ml of blood was prepared.

#### Laparoscopic surgery

**Fig. 2:**
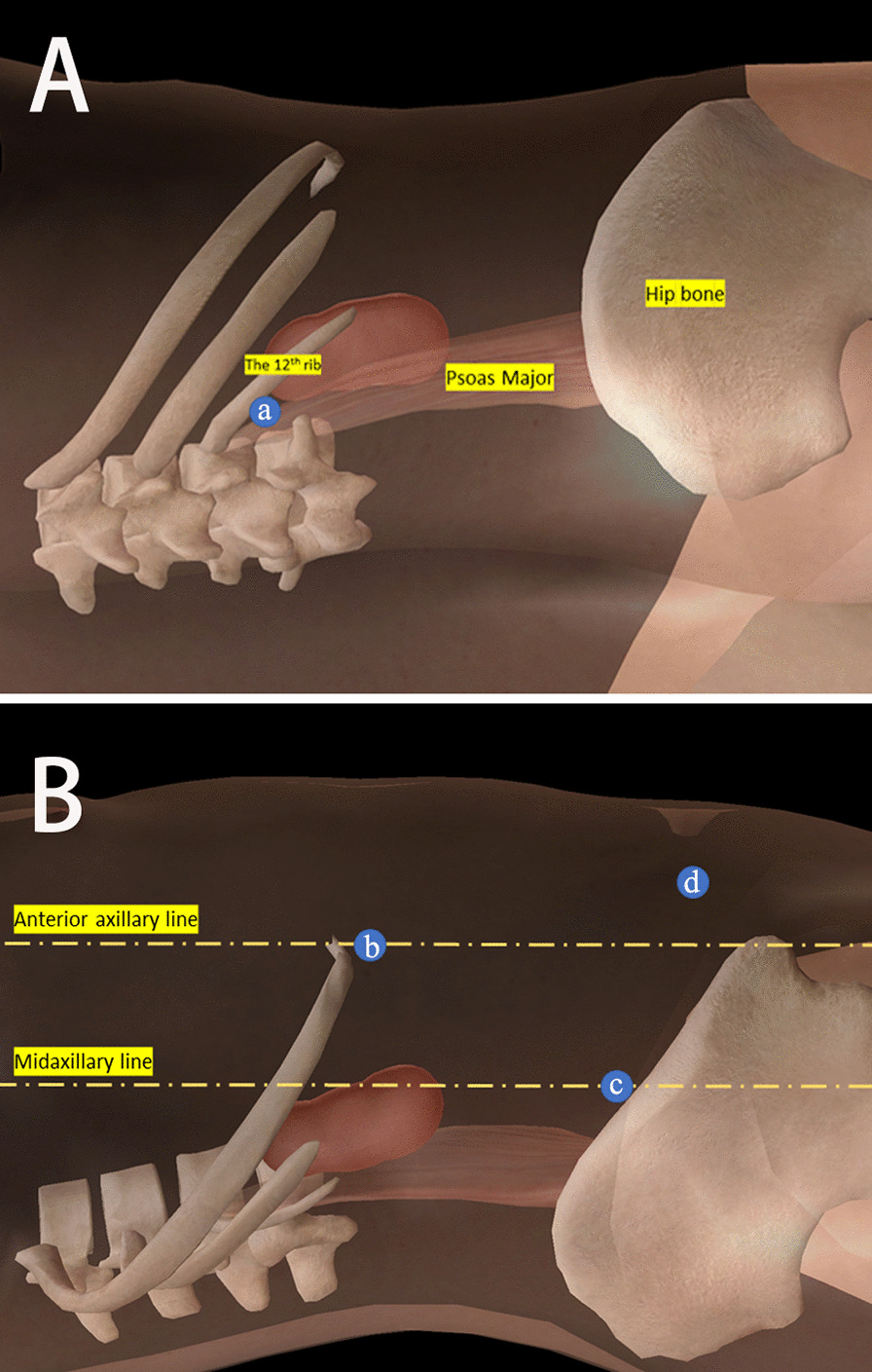
The Trocars placement. Four Trocars were placed at the intersection of the twelfth rib and the edge of the psoas major (a), the costal margin along the anterior axillary line (b), the iliac crest along the midaxillary line (c), 5 cm superior medial to iliac crest (d), respectively. **A** posterior view; **B** lateral view

Three patients underwent retroperitoneal laparoscopic surgery. The ports were set at the following points to introduce the camera and tools (Fig. 2): the intersection of the twelfth rib and the edge of the psoas major, the costal margin along the anterior axillary line, and the iliac crest along the midaxillary line. Trocars of 12, 12, and 11 mm were placed. A 5 mm assisting port was introduced 5 cm medial and superior to the superior iliac spine when necessary. The pneumoperitoneum pressure was maintained at 12 mmHg.

First, the ureter and IVC were found at the lower pole of the right kidney and carefully separated upwards along the IVC. The following steps were different among cases. (1) In case 1, the IVC was dissociated from the upper right renal pole because the mass was located in the adrenal area. Upon separating the dorsal and ventral sides of the tumor, the adrenal gland was found to be significantly compressed by the solid mass, while there was still space between them. Then, the right kidney was dissociated and pressed down, and we found that the upper pole of the right kidney had no contact with the lower tumor boundary. However, the tumor was adhered to the adrenal gland so tightly that part of the adrenal gland was excised after clipping the blood vessels with Hem-o-loks. Further separation revealed that the tumor remarkably invaded and compressed the IVC, indicating that the IVC was the primary cancerous site. Therefore, the Satinsky clamp was placed to partially block the bloodstream, and the tumor was removed entirely. The venous incision was closed with 4–0 Prolene suture. (2) In case 2, the tumor was located in the right renal hilum, and the ventral side of the tumor could be identified when the IVC was separated to the level of the RV. The lower pole and the dorsal and ventral sides of the kidney were fully dissociated, followed by lifting the lower renal pole to fully separate the tumor on the ventral side. It was found that the tumor partially invaded the RV wall, so a more extended segment of the IVC was dissociated lengthwise to prepare for blocking, and then the surgery was converted to an open approach. We made a Chevron incision to open the abdominal cavity and blocked the RV with a bulldog clamp. The Satinsky clamp was used to partially block the IVC at the renal level. Then, we cut open the RV and removed the mass entirely. After the defect of the RV and IVC was closed with 4–0 Prolene suture, the bloodstreams of the IVC, renal artery, and RV were recovered. (3) In case 3, the tumor was also located in the adrenal area. During the operation, however, it was found that the adrenal gland was normal in shape without cancerous features, and the tumor was lobulated and inserted into the IVC locally, suggesting that the solid mass invaded the IVC directly to form a tumor thrombus without extending through the central AV. Therefore, we blocked the IVC at the site inferior to the tumor thrombus with the method described in case 1, followed by increasing the pneumoperitoneum pressure to 20 cmH_2_O, and tumor thrombectomy was performed.

#### Open surgery

One patient (case 4) underwent open surgery. We chose the Chevron incision and extended it by 10 cm toward the base of the xiphoid process. Kocher's method was used to expose the retroperitoneal cavity, which revealed that the tumor was firmly adhered to the right kidney and wrapped around the right renal artery. Therefore, we decided to remove the tumor together with the right kidney. The liver-IVC and kidney-IVC gaps were dissociated, and the ureter was ligated and cut off 7 cm inferior to the kidney. Multiple renal arteries adjacent to the dorsal side of the IVC were exposed, ligated, and cut off after flipping up the tumor and the kidney. The adrenal gland was retained because no abnormalities were detected in the adrenal area. It was found that the tumor thrombus extended to the retrohepatic IVC. Therefore, the liver was dissociated and flipped up to expose the IVC further by cutting off the surrounding ligaments. Blood vessels were blocked in the following order: the distal end of the IVC below the RV, the left RV, the first hepatic hilum and, finally, the proximal end of the IVC; the IVC was then cut open to remove the tumor, right kidney, and tumor thrombus. The IVC incision was sutured with 4–0 Prolene. In the end, the bloodstream was recovered by releasing the proximal end of the IVC, the left RV, and the distal end of the IVC in order.

### Follow-up

The patients were followed up for the first time one month after the operation, every three months in the first two years, every six months after two years. Follow-up was carried out by outpatient revisit and phone call.

## Results

As shown in Table 1, among the four patients studied, one demonstrated local and systemic discomfort before surgery, and the remaining patients were asymptomatic. The hormone tests of the kidney and adrenal gland showed no abnormalities, and the preoperative ASA evaluation demonstrated grade 2.

The surgeries of the four patients were completed, and no perioperative deaths occurred. The median duration of the operation was 314.5 (min–max = 224–467) min. The tumors were entirely resected, with a median size of 7.45 (min–max = 4.2–10.7) cm. The median amount of intraoperative bleeding was 550 (min–max = 200–1500) ml. In cases 1, 3, and 4, intraoperative transfusion of red blood cells was 800 ml, 2000 ml, and 800 ml, respectively. In addition, in case 3, 1400 ml of plasma transfusions was received. The median postoperative hospitalization ranged from 7 (min–max = 6 -12) days. In case 1, a Clavien-Dindo II complication was reported [6]: postoperative retroperitoneal bleeding with a decrease in hemoglobin to 77 g/L on the third day after the operation. With the use of abdominal bandage compression and the transfusion of 2 U of suspended red blood cells and 400 ml of plasma, the hemoglobin level returned to normal, and no further surgery was needed. One week after the operation, the median serum creatinine level was 85 (min–max = 70–99) µmol/L.

**Fig. 3:**
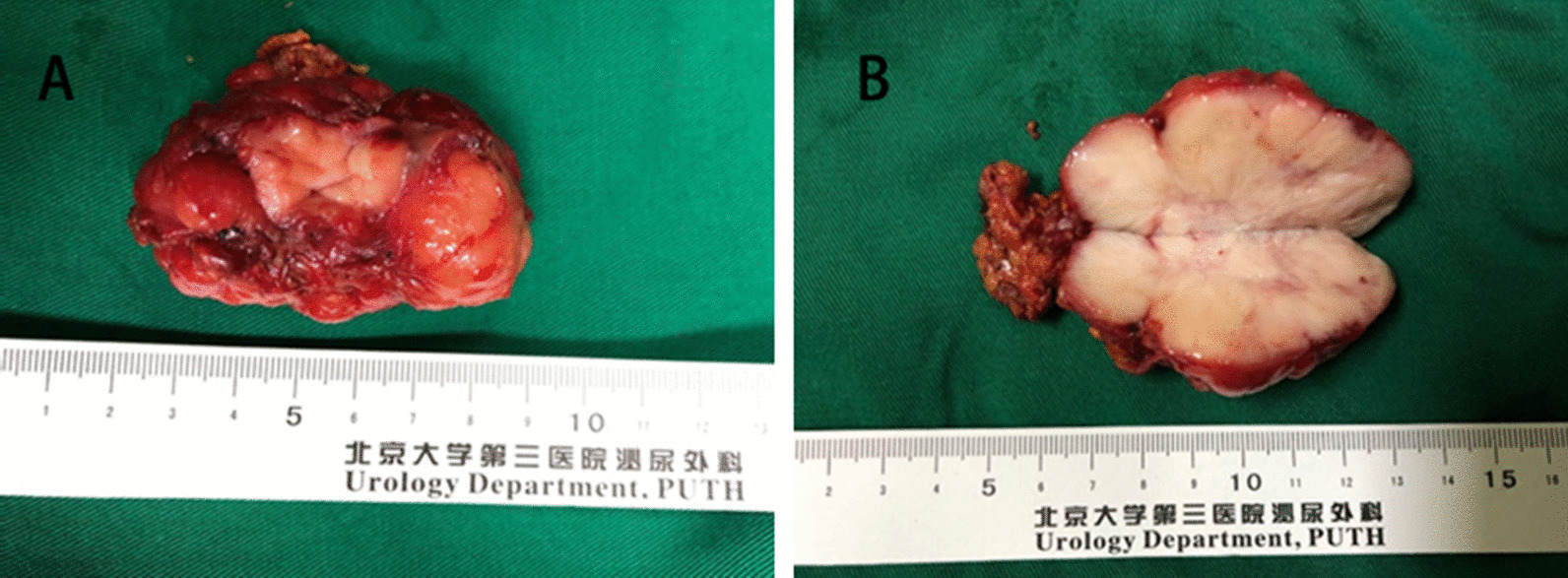
Gross findings of the tumor (Case 1). The irregular solid mass was covered with a capsule (**A**). The cut surface was greyish-white, and focal necrosis was detected (**B**)

**Fig. 4:**
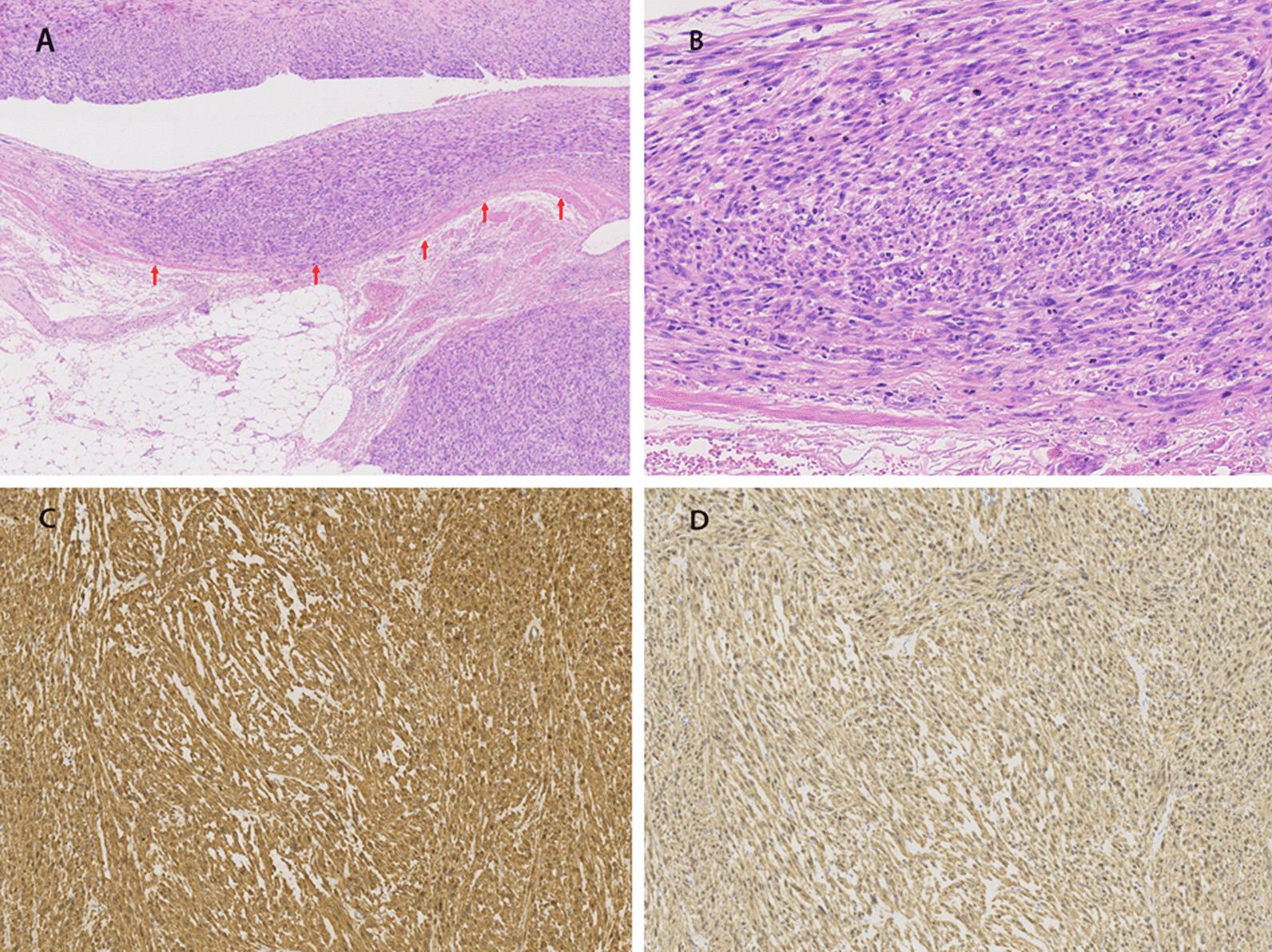
The histological examination of RVLMS. **A** Renal LMS arose from renal vein (red arrow), 5×; **B** Renal LMS exhibited high mitotic activity and cytologic atypia, 200×; **C**, **D** Immunohistochemistry. Renal LMS expressed SMA (**C**) and Desmin (**D**), 100×

The pathological examination showed the all solid tumors was irregular in shape with negative margins (Fig. 3A), the cut surface was greyish-white, and the outer part of the mass was covered with a capsule (Fig. 3B). Spindle-shaped cells with high mitotic activity and cytologic atypia were detected (Fig. 4A, B), and immunochemical test showed SMA ( +) and Desmin ( +) (Fig. 4C, D). We used the French Federation of Cancer Centers (FNCLCC) system to evaluate the histological performance of four cases [7], and showed Case 1 and 3 were classified as grade G1, and case 2 and 4 were classified as G2.

At the last follow-up, all patients were still alive, and the median follow-up time was 16 (min–max = 13–21) months. In case 1, local recurrence without symptoms was reported during the review 15 months after the operation. The patient was recommended to receive radiotherapy but refused. The remaining three patients had no recurrence or metastasis.

## Discussion and literature review

LMS is a common histological type of retroperitoneal sarcoma, but only 5% of cases are reported to have a vascular origin, of which more than 70% arise from the IVC, followed by the renal vein, central adrenal vein, iliac vein, etc [8, 9]. The disease is often diagnosed in middle-aged and older adults, and 80% of patients are female [10]. The etiology is still unclear and may be related to endocrine misregulation. Carvalho et al. reported a 78-subject cross-sectional study and found that patients with RVLMS were more likely to develop uterine fibroids or endocrine tumors [11]. The absence of critical manifestations and biomarkers as well as the difficulty of imaging diagnosis leads to a less accurate preoperative diagnosis.

### Comprehensive preoperative evaluation and preparation are necessary

No guidelines currently provide clear diagnostic criteria for RVLMS. Preoperative diagnosis, which is still challenging, is based on the comprehensive evaluation of clinical manifestations and imaging studies.

The most common presentation of RVLMS is abdominal discomfort, edema of the lower extremities, or weight loss, which is often caused by the obstruction of venous return due to tumor compression. For RVLMS originating in the IVC, symptoms are related to the site invaded. In many studies, the IVC is divided into upper, middle, and lower segments according to the level of the renal hilum and hepatic vein [4, 12, 13]. Lower RVLMS mainly presents as edema or numbness of the lower extremities; middle RVLMS patients may suffer from right upper quadrant abdominal or back pain. Ascites, Budd-Chiari syndrome, or even right heart failure may be present in upper RVLMS [4, 14]. Federico et al. suggested that most cases of RVLMS originate from the lower segment (37–42%) or middle segment (43–50.8%) of the IVC [13]. However, due to the slow growth rate, the large IVC lumen, and the possibility of growing in opposition to the lumen, only a few patients have shown positive manifestations. In this study, case 1 was consistent with middle and lower RVLMS, which was consistent with the imaging and intraoperative findings.

In comparison, RVLMS originating in the renal vein is much rarer. Aguilar et al. conducted a literature review and found only 29 cases, and nearly 60% of cases had lesions in the left renal vein. Nonspecific symptoms such as abdominal or lower back pain will appear only when the tumor infiltrates into surrounding tissues or the renal veins [15]. Physical examination usually shows no positive signs, and renal percussive pain can occasionally be found. Therefore, diagnosis based on clinical manifestations is very challenging. In this study, cases with this presentation were scarce.

Imaging plays a critical role in RVLMS diagnosis. In this study, the key findings were as follows. (1) The vessels involved (IVC or RV) were significantly dilated. (2) Intra- or extraluminal tumors were unevenly enhanced by CECT. (3) Massive collateral circulations were seen in patients with blocked vessels. These findings are consistent with the LMS imaging appearance described by Mu et al. in 2011 [16]. Federico et al. found that the best view was obtained during the portal venous phase, which provided sufficient details to evaluate the tumor growth pattern and infiltration [9, 13]. It is of great importance for differential diagnosis.

Liposarcoma is the most common retroperitoneal tumor with more insidious manifestations, and the tumor composition are related to the histological differentiation, which is similar to the situation of RVLMS and easily misdiagnosed [17]. It is not difficult to distinguish well-differentiated liposarcoma from RVLMS by clear boundaries, large fat droplets, and few soft tissues. However, it is a different story for high-grade, poorly differentiated liposarcoma because of the fuzzy edge and lack of fat [13, 17, 18]. Calcification may be present in 30% of lipomyosarcoma, which provides clues for identification [17].

Neurogenic tumors, which are common among teenagers, are another disease worthy of consideration. Neurofibroma has a clear boundary and uniform enhancement in CECT, and target signs can occasionally be identified. Schwannomas are generally located next to the spinal cord and do not invade blood vessels. However, as tumors grow substantially, necrosis and uneven enhancement may occur, making identification difficult [19–22].

Notably, the differential diagnosis of paraganglioma is meaningful for treatment. Patients with paraganglioma often show sympathetic symptoms, such as palpitation, higher blood pressure and, sometimes, hypertensive crisis due to a high level of catecholamines [16, 21, 23]. Tumor size, morphology, and central necrosis can simulate RVLMS [17]. Moreover, a recent report of silent paraganglioma, which can be stimulated intraoperatively and lead to uncontrollable hypertension, revealed that the differential diagnosis between RVLMS and paraganglioma is more urgent and difficult [23, 24]. Therefore, in this study, except for in case 4 where the diagnosis of LMS was made preoperatively by biopsy, patients were asked to take phenoxybenzamine for two weeks before the operation to achieve relatively low blood pressure to prevent hypertensive crisis caused by potential misdiagnosis.

Preoperative pathologic biopsy is one of the gold standards for the diagnosis of RVLMS, especially in cases difficult to distinguish from unresectable tumors, such as lymphoma [25]. This method is also recommended for suspicion of metastasized tumors. However, many factors, including tumor location, adjacent organs, blood vessels, and patients' willingness, affect the feasibility and accuracy of preoperative biopsy, so this study did not include it as a routine examination.

### MDT-guided surgical resection regimen

Due to the particularity of retroperitoneal tumors in terms of biological characteristics, pathological types, occurrence locations, and lack of effective treatment methods, multidisciplinary team (MDT) collaboration focuses on individualized treatment is critical.

Although limited cases reported, *en bloc* resection with a negative margin is still supposed to be the core of treatment [26]. Gauzel et al. pointed that patients who undergo radical resection of RVLMS tend to have a better prognosis, while the positive margins imply a higher recurrence rate, and more challenging to perform reoperation. Repeated recurrence significantly reduces the long-term survival rate [27–29].

Neoadjuvant chemotherapy can exert a survival benefit on most soft tissue sarcomas. However, the LMS is less sensitive to it, so it is not routinely performed, except for several situations: (1) Preoperative imaging shows extensive reaction areas around the tumor, suggesting that the tumor is more aggressive and infiltrating. (2) Biopsy shows tumors cells has high malignant potential, such as high mitotic activity, atypia, or high Ki-67 expression. (3) Recurrent tumors. Chemotherapy has achieved a certain effect, but further rigorous clinical research is still needed.

The surgical plan should be individualized according to the patient's condition, tumor location, and adjacent blood vessels and organs. The four operations' common points are (1) exposure of the retroperitoneum and involved tissues and protection of the ureter; (2) identification of vital blood vessels in the surgical field and determination of the lesion; (3) careful dissociation of the masses; (4) blockage of the bloodstream of the involved vessels (IVC or renal pedicle) with vascular block forceps or a Satinsky clamp; (5) complete removal of the tumor, as well as affected organs and tumor thrombus, where the milking method can be considered to decrease the level of tumor thrombus [30, 31]; and (6) open blood flow after vessel reconstruction.

Bloodstream blockage should be addressed during surgery, especially for IVC masses. Although the collateral circulation may compensate for the reduction in venous return, IVC blockage is still a challenge for the patient's cardiac function when using traditional vessel-blocking forceps, which entirely block the bloodstream. Wachtel et al. conducted a 6-patient case series of IVC RVLMS and proposed the use of venous-venous shunts, that is, the establishment of shunts at both ends of the blocked blood vessels by pumps to maintain stable cardiac blood volume [5]. In our study, we innovatively used the Satinsky clamp to partially block the IVC that was invaded by the tumor but did not completely block the blood flow, followed by removing and reconstructing the affected blood vessel wall. The remaining IVC blood flow and collateral circulation ensured that the return blood volume was relatively sufficient, which was more convenient than a veno-venous shunt. There is no research that has compared the above two methods statistically, and for patients whose tumors originate from shorter blood vessels where the Satinsky clamp cannot be placed (for example, the renal vessels in case 2), nephrectomy may still be considered [5, 19].

Interestingly, for patients with contralateral renal insufficiency, solitary kidneys, and a strong willingness to retain the kidneys, it is also possible to undergo conversion to open surgery to remove the tumor and perform vascular reconstruction. Nevertheless, the scope is small, and the operation is hard to perform, making the risk of recurrence and bleeding after surgery much higher than that of radical nephrectomy [10, 32]. Therefore, it is necessary to integrate the patient's condition and willingness to communicate with the patients and their family members and to reasonably select the operation strategy.

RVLMS is highly malignant and has a poor prognosis. According to current literature reports, tumors more than 3 cm in diameter, positive margins, and kidney-sparing surgery are risk factors for local recurrence and distant metastasis. Aguilar et al. analyzed 30 cases reported in the literature and found that 30% of patients had no relapse or metastasis (mean follow-up of 78 months), 23% of patients had local recurrence and metastasis and were still alive, and 37% of patients died after relapse (mean follow-up of 48 months) [15].

### Postoperative adjuvant therapy

The adjuvant chemotherapy of LMS is controversial. Some randomized studies have shown that adjuvant chemotherapy can improve disease-free survival (DFS). For example, the Italian Sarcoma Collaborative Group adopted the EI regimen for primary large adult soft tissue sarcoma, and showed that the chemotherapy group obtained nearly 30 months of DFS and OS prolongation compared with the non-chemotherapy group [33]. However, SMAC meta-analysis, which included four large randomized controlled trials involving more than 1900 patients, showed no difference in OS between patients receiving postoperative monotherapy and those without chemotherapy [28, 34]. Therefore, the specific population should be selected for adjuvant chemotherapy, and the increase of dose and combined medication should be considered. The adjuvant chemotherapy is recommended with the following conditions: (1) high grade and a large volume of tumor; (2) The operation did not reach the safe surgical boundary; (3) Recurrent tumor operations.

At present, Doxorubicin combined with Ifosfamide is still an ideal chemotherapy regimen. A clinical trial done by The European Organization for Research and Treatment of Cancer (EORTC) (No.62012, NCT00061984) showed that the response rate (26 vs 14%), progression-free survival (7.4 vs 4.6 months) and overall survival (14.3 vs 12.8 months) of combined medication were better than those of Doxorubicin alone [35, 36]. However, there is no recognized second-line regimens, and different drugs should be selected according to the histological type.

The application of adjuvant radiotherapy has not reached a consensus, most clinical studies show optimistic prospects, but waiting for more rigorous clinical trials to prove its effectiveness [37]. In this study, the follow-up imaging examination of case 1 showed local recurrence. The recurrent tumor bed was located in the operation area, and the patient refused to receive systemic chemotherapy, so local radiotherapy was recommended. No progression was found in the following 2 months. Unfortunately, the patient was lost to follow-up and could not be further evaluated.

Postoperative adjuvant targeted therapy is also worth exploring in the future. In recent years, a number of clinical trials of targeted medications for LMS are ongoing and showed optimistic results. In 2016, FDA accelerated the approval of Olaratumab (PDGFRα antibody) is on the market, becoming the first first-line drug for soft tissue sarcoma [38, 39].

In terms of limitations, the nature of this case series study in terms of the lack of a control group made the results only a reference for clinical practice rather than a guideline. Due to the low prevalence of RVLMS, there remains no consensus on the standards for treatment of the disease. Case-controlled or cohort studies should be conducted to determine the potential cause and prognostic factors in future work.

## Conclusion and perspective

This study summarizes the diagnosis and treatment experience of RVLMS in our center based on data from four patients. RVLMS is complicated to diagnose preoperatively due to uncharacteristic manifestations and confusing imaging results. Most of the patients were diagnosed by intraoperative and postoperative pathology. Completed preoperative evaluation and MDT collaboration are essential, as well as careful designation of the surgical plan to remove the mass and rebuild the blood vessels. The innovative use of the Satinsky clamp in our center to block blood vessels reduced the difficulty of surgery, but we could not statistically compare this method with the traditional blocking method or with veno-venous shunting because of the small number of cases. In short, to raise the concern of RVLMS and facilitate guideline establishment, multicenter research is required to collect enough cases and obtain more powerful and persuasive conclusions to guide future treatment.

## Data Availability

The datasets generated during and/or analysed during the current study are not publicly available due to some prognostic data are the key findings of on-going further study but are available from the corresponding author on reasonable request.

## Abbreviations

RVLMS: Retroperitoneal vascular leiomyosarcoma
IVC: Inferior vena cava
RV: Renal vein
AV: Adrenal vein
ASA: American Society of Anesthesiologists
CTU: Computed tomography urography
VMA: 3-Methyl-4-hydroxymandelic acid
ACTH: Adrenocorticotropic hormone
RAAS: Renin-angiotensin II-aldosterone system
CECT: Contrast-enhanced computed tomography
MDT: Multidiscipline team
LMS: Leiomyosarcoma
